# Supplementary material for: Characterization of Staphylococcus lugdunensis biofilm reveals key differences according to clonal lineage and iron availability
Source: Biofilm. 2025 Nov 6;10:100329. doi: 10.1016/j.bioflm.2025.100329 (PMC12648618; doi:10.1016/j.bioflm.2025.100329)
Supplement: Multimedia component 1 [file mmc1.docx]

Supplementary Material

Characterization of *Staphylococcus lugdunensis* biofilm reveals key differences according to clonal lineage and iron availability

## Supplementary Tables

**Supplementary Table 1.** Characteristics and levels of biofilm production of the 49 clinical strains of *S. lugdunensis* studied. ND: No Data, -: no production of biofilm, +: weak production, ++: moderate production, +++: strong production, TSBG: Trypticase Soy Broth supplemented with 1% of glucose, RPMI: RPMI supplemented with 1% of casamino acids.

| **Strain** | **Clonal complex (CC)** | **Sequence typing (ST)** | **City** | **Clinical sources / sampling** |  | **Date of collection** |  |  |
| --- | --- | --- | --- | --- | --- | --- | --- | --- |
|  |  |  |  |  | **Device (Y) or not (N)** |  | **Biofilm production**  **(TSBG)** | **Biofilm production**  **(RPMI)** |
| 57FJM | 1 | 1 | Strasbourg | Infection | N | 2015 | + | ++ |
| SL_29 | 1 | 1 | Rouen | Infection | Y | 2007 | ++ | ++ |
| SL_122 | 1 | 1 | Tours | Carriage | N | ND | +++ | ++ |
| 37BH | 1 | 1 | Strasbourg | Infection | N | 2014 | +++ | + |
| SL_117 | 1 | 1 | Kronoberg | Carriage | ND | ND | +++ | ++ |
| 07SM | 1 | 6 | Strasbourg | Infection | N | 2014 | ++ | +++ |
| 33RM | 1 | 6 | Strasbourg | Infection | N | 2014 | +++ | ++ |
| SL_13 | 1 | 6 | Rouen | Infection | ND | 2001 | +++ | ++ |
| SL_74 | 1 | 12 | Nancy | Infection | N | ND | +++ | ++ |
| C60 | 1 | 12 | Strasbourg | Carriage | Y | 2014 | ++ | +++ |
| 27BC | 1 | 12 | Strasbourg | Infection | Y | 2014 | +++ | - |
| 74KR | 2 | 2 | Strasbourg | Infection | N | 2015 | +++ | + |
| SL_85 | 2 | 2 | Montpellier | Infection | Y | 2011 | +++ | ++ |
| SL_56 | 2 | 14 | Nantes | Infection | N | ND | +++ | + |
| SL_73 | 2 | 19 | Nancy | Infection | N | ND | +++ | + |
| 64865028 | 2 | ND | Rouen | Carriage | ND | 2016 | +++ | ++ |
| C47 | 3 | 3 | Strasbourg | Carriage | Y | 2014 | +++ | ++ |
| C85 | 3 | 3 | Strasbourg | Carriage | N | 2015 | + | +++ |
| 50SD | 3 | 3 | Strasbourg | Infection | Y | 2015 | + | +++ |
| 31FE | 3 | 3 | Strasbourg | Infection | N | 2014 | +++ | ++ |
| C08 | 3 | 3 | Strasbourg | Carriage | N | 2013 | + | +++ |
| C27 | 3 | 3 | Strasbourg | Carriage | N | 2014 | ++ | +++ |
| 03MC | 3 | 3 | Strasbourg | Infection | N | 2013 | +++ | + |
| 04SZ | 3 | 3 | Strasbourg | Infection | Y | 2013 | +++ | ++ |
| 76LE | 3 | 3 | Strasbourg | Infection | N | 2015 | ++ | ++ |
| 16060354 | 3 | ND | Rouen | Carriage | ND | 2016 | + | +++ |
| 83815155 | 3 | 3 | Rouen | Infection | Y | 2018 | +++ | + |
| 34SM | 4 | 4 | Strasbourg | Infection | N | 2014 | ++ | + |
| 48SA | 4 | 4 | Strasbourg | Infection | N | 2015 | ++ | - |
| SL_62 | 4 | 17 | Bordeaux | Infection | N | ND | + | ++ |
| 16084449 | 4 | ND | Rouen | Infection | ND | 2016 | + | ++ |
| 25AC | 5 | 5 | Strasbourg | Infection | Y | 2014 | - | ++ |
| 73KM | 5 | 5 | Strasbourg | Infection | Y | 2015 | +++ | + |
| SL_66 | 5 | 18 | Bordeaux | Infection | N | ND | ++ | ++ |
| 16035553 | 5 | ND | Rouen | Infection | ND | 2016 | - | ++ |
| 16103574 | 5 | ND | Rouen | Infection | ND | 2016 | ++ | +++ |
| SL_118 | 6 | 10 | Kronoberg | Carriage | ND | ND | + | +++ |
| SL_55 | 6 | 10 | Nantes | Infection | ND | ND | ++ | +++ |
| SL_DSM 4804 | 6 | 24 | Lyon | Infection | N | ND | ++ | +++ |
| 22FJ | 6 | 24 | Strasbourg | Infection | N | 2014 | ++ | ++ |
| 16084028 | 6 | ND | Rouen | Infection | ND | 2016 | ++ | +++ |
| 77HJ | 7 | 26 | Strasbourg | Infection | Y | 2015 | ++ | ++ |
| C42 | 7 | 26 | Strasbourg | Carriage | Y | 2014 | ++ | +++ |
| 70GR | 7 | 27 | Strasbourg | Infection | Y | 2015 | +++ | - |
| 16043959 | 7 | ND | Rouen | Infection | N | 2016 | +++ | ++ |
| SL_10 | Singleton | 13 | Rouen | Infection | Y | 2000 | ++ | ++ |
| 65KN | Singleton | 28 | Strasbourg | Infection | N | 2015 | + | ++ |
| 69MP | Singleton | 28 | Strasbourg | Infection | N | 2015 | + | ++ |
| 16116446 | Singleton | ND | Rouen | Carriage | ND | 2016 | + | - |

**Supplementary Table 2.** List of the 321 proteins identified in the biofilm matrix of *S. lugdunensis* 03MC and SL-55 strains. Proteins showing a *p* value > 0.05 or a power < 0.8 are in grey. Proteins with unchanged abundance between the two strains are in green. Significantly more abundant proteins in 03MC are in blue and those of SL-55 in orange.

| **Accession number RefSeq** | **Accession number KEGG** | **Description** | **Mass** | **Normalized abundance 03MC** | **Normalized abundance SL-55** | **Max fold change** |
| --- | --- | --- | --- | --- | --- | --- |
| WP_002479331.1 | slg:SLGD_02063 | thioredoxin-disulfide reductase | 33602 | 4085,48127 | 4473,36326 | 1,09494157 |
| WP_002458821.1 | slg:SLGD_01180 | D-amino-acid transaminase | 31931 | 6932,06599 | 6895,72191 | 1,00527053 |
| WP_002478224.1 | slg:SLGD_01520 | thymidylate synthase | 36781 | 8620,94849 | 8273,27112 | 1,04202417 |
| WP_002478537.1 | slg:SLGD_00364 | CHAP domain-containing protein | 27154 | 15338,3466 | 15497,9468 | 1,01040531 |
| WP_002433901.1 | slg:SLGD_01496 | cold-shock protein CspA | 7333 | 8205,61031 | 6876,98316 | 1,19319913 |
| WP_002479038.1 | slg:SLGD_00229 | dihydrolipoyl dehydrogenase | 48244 | 5909,24691 | 5861,77974 | 1,00809774 |
| WP_002477954.1 | slg:SLGD_01035 | ferritin | 19585 | 7837,80198 | 8202,02863 | 1,04647051 |
| WP_002460280.1 | slg:SLGD_00598 | nitrate reductase subunit beta | 59248 | 2000,641 | 2347,12142 | 1,1731847 |
| WP_002478333.1 | slg:SLGD_01725 | division/cell wall cluster transcriptional repressor MraZ | 17226 | 26899,9449 | 29028,0238 | 1,0791109 |
| WP_002459290.1 | slg:SLGD_01640 | GTP-sensing pleiotropic transcriptional regulator CodY | 28816 | 27982,0419 | 28075,1373 | 1,00332697 |
| WP_002478011.1 | slg:SLGD_01172 | leucine--tRNA ligase | 91856 | 10342,4088 | 11310,8307 | 1,09363601 |
| WP_002478015.1 | slg:SLGD_01179 | dipeptidase PepV | 52641 | 12015,4897 | 11578,6766 | 1,03772565 |
| WP_002478372.1 | slg:SLGD_01791 | phosphoenolpyruvate--protein phosphotransferase | 63541 | 21083,0539 | 20572,9743 | 1,02479367 |
| WP_002479388.1 | slg:SLGD_02160 | LysM peptidoglycan-binding domain-containing protein | 28387 | 51885,4312 | 45654,594 | 1,13647777 |
| WP_012990851.1 | slg:SLGD_01537 | 4-oxalocrotonate tautomerase | 6730 | 7079,11099 | 9438,99505 | 1,33335882 |
| WP_002479220.1 | slg:SLGD_02519 | LacI family transcriptional regulator | 36994 | 6002,92199 | 6493,18798 | 1,08167122 |
| WP_002459661.1 | slg:SLGD_02021 | organic hydroperoxide resistance protein | 15327 | 993,069178 | 1039,41316 | 1,04666743 |
| WP_002460790.1 | slg:SLGD_00196 | hypothetical protein | 56508 | 57889,2934 | 62306,7661 | 1,07630898 |
| WP_002461227.1 | slg:SLGD_00920 | F0F1 ATP synthase subunit beta | 51472 | 111764,61 | 126687,196 | 1,13351798 |
| WP_002478566.1 | slg:SLGD_00399 | acetolactate synthase AlsS | 60562 | 5084,59566 | 5658,03884 | 1,11278049 |
| WP_002479000.1 | slg:SLGD_00283 | linear amide C-N hydrolase | 36823 | 1554,03497 | 1666,69202 | 1,07249325 |
| WP_002478041.1 | slg:SLGD_01221 | alanine dehydrogenase | 40167 | 16582,1868 | 18154,1876 | 1,09480057 |
| WP_002478592.1 | slg:SLGD_00438 | hypothetical protein | 18049 | 2425,36412 | 3132,20484 | 1,29143695 |
| WP_002479341.1 | slg:SLGD_02076 | autolysin | 29626 | 229637,9 | 469340,275 | 2,04382759 |
| WP_002458966.1 | slg:SLGD_01322 | HD domain-containing protein | 22142 | 2057,26455 | 2246,32004 | 1,09189654 |
| WP_002461229.1 | slg:SLGD_00919 | F0F1 ATP synthase subunit gamma | 31908 | 13696,3055 | 12317,2619 | 1,11196024 |
| WP_002458764.1 | slg:SLGD_01115 | transcriptional regulator | 17782 | 3094,42175 | 3551,07205 | 1,14757209 |
| WP_002461230.1 | slg:SLGD_00918 | F0F1 ATP synthase subunit alpha | 54607 | 85621,0185 | 103711,17 | 1,21128166 |
| WP_002478201.1 | slg:SLGD_01478 | oligoendopeptidase F | 69058 | 4155,05391 | 2945,49169 | 1,41064866 |
| WP_002478848.1 | slg:SLGD_00822 | antibiotic biosynthesis monooxygenase | 25413 | 343,784743 | 268,774124 | 1,27908423 |
| WP_002460281.1 | slg:SLGD_00597 | nitrate reductase subunit alpha | 139173 | 5193,56632 | 7543,95281 | 1,45255733 |
| WP_002459622.1 | slg:SLGD_01979 | cystatin-like fold lipoprotein | 16387 | 1726,03319 | 1353,90194 | 1,27485835 |
| WP_002478272.1 | slg:SLGD_01620 | polyribonucleotide nucleotidyltransferase | 76961 | 57303,6557 | 44276,107 | 1,29423429 |
| WP_002478360.1 | slg:SLGD_01768 | DUF1054 domain-containing protein | 23947 | 42781,9605 | 67356,4218 | 1,57441176 |
| WP_002478284.1 |  | type I DNA topoisomerase | 79268 | 14791,7238 | 26403,3671 | 1,78500947 |
| WP_000387527.1 | slg:SLGD_00777 | 50S ribosomal protein L22 | 12827 | 61264,7803 | 92444,7756 | 1,50893834 |
| WP_002459288.1 | slg:SLGD_01638 | elongation factor Ts | 32257 | 71182,2201 | 58199,2122 | 1,22307876 |
| WP_002479296.1 | slg:SLGD_01974 | type VII secretion protein EsaA | 114771 | 272,673172 | 426,809392 | 1,56527828 |
| WP_002460922.1 |  | heme uptake protein IsdC | 24638 | 19694,1728 | 39014,5723 | 1,98102112 |
| WP_002459784.1 | slg:SLGD_02142 | MarR family transcriptional regulator | 16816 | 5614,88493 | 4210,34895 | 1,33359135 |
| WP_002478149.1 | slg:SLGD_01404 | phosphogluconate dehydrogenase (NADP(+)-dependent, decarboxylating) | 51854 | 27574,0563 | 42377,6604 | 1,53686712 |
| WP_002435373.1 | slg:SLGD_01746 | 50S ribosomal protein L32 | 6497 | 108217,344 | 139702,225 | 1,29094118 |
| WP_002479137.1 | slg:SLGD_00094 | YSIRK signal domain/LPXTG anchor domain surface protein | 71495 | 22169,9429 | 16636,0155 | 1,3326474 |
| WP_002459006.1 | slg:SLGD_01362 | superoxide dismutase | 22957 | 54312,4832 | 42734,1712 | 1,27093802 |
| WP_002461333.1 | slg:SLGD_01045 | DNA-binding response regulator | 23643 | 386,158658 | 201,44152 | 1,9169765 |
| WP_002478014.1 | slg:SLGD_01178 | YtxH domain-containing protein | 15214 | 5871,31551 | 3696,80865 | 1,58821191 |
| WP_002477941.1 | slg:SLGD_01018 | adenylosuccinate lyase | 49822 | 13873,2795 | 9282,04443 | 1,49463619 |
| WP_002461308.1 | slg:SLGD_01066 | thioredoxin-dependent thiol peroxidase | 17336 | 5715,39013 | 4349,25781 | 1,314107 |
| WP_002478830.1 | slg:SLGD_00773 | 50S ribosomal protein L4 | 22416 | 5077,37713 | 3707,64893 | 1,36943309 |
| WP_002459651.1 | slg:SLGD_02011 | MetQ/NlpA family ABC transporter substrate-binding protein | 30141 | 4373,26265 | 3509,72484 | 1,24604145 |
| WP_002459935.1 |  | DNA-directed RNA polymerase subunit beta' | 135172 | 4848,56927 | 3460,42006 | 1,40115049 |
| WP_002479202.1 | slg:SLGD_02547 | DNA polymerase III subunit beta | 41914 | 9957,63207 | 13262,572 | 1,33190019 |
| WP_001118667.1 | slg:SLGD_00771 | 30S ribosomal protein S10 | 11569 | 40029,5427 | 24175,1526 | 1,65581344 |
| WP_002478654.1 | slg:SLGD_00519 | exopolyphosphatase | 58338 | 7481,01449 | 10736,6862 | 1,43519121 |
| WP_002459515.1 | slg:SLGD_01872 | tryptophan--tRNA ligase | 37125 | 3345,08925 | 2311,85422 | 1,44692914 |
| WP_002460053.1 | slg:SLGD_00790 | 50S ribosomal protein L30 | 6550 | 1723,2212 | 1246,93421 | 1,38196642 |
| WP_002478597.1 | slg:SLGD_00446 | hydroxymethylglutaryl-CoA synthase | 43549 | 15436,1636 | 11442,3087 | 1,34904276 |
| WP_002459951.1 | slg:SLGD_02361 | glutamate--tRNA ligase | 56233 | 17786,9081 | 12176,3375 | 1,46077653 |
| WP_002479305.1 | slg:SLGD_02017 | arsenate reductase | 13583 | 26853,5981 | 14505,318 | 1,85129331 |
| WP_002478659.1 | slg:SLGD_00529 | DNA-binding protein | 54586 | 11562,5301 | 9302,46021 | 1,24295399 |
| WP_002460052.1 | slg:SLGD_00791 | 50S ribosomal protein L15 | 15524 | 13225,7374 | 6705,49725 | 1,97237236 |
| WP_002479297.1 | slg:SLGD_01976 | CHAP domain-containing protein | 32913 | 12558,7006 | 6376,29923 | 1,96959084 |
| WP_002459936.1 | slg:SLGD_02347 | DNA-directed RNA polymerase subunit beta | 133273 | 6527,05831 | 10249,3872 | 1,57029197 |
| WP_002460060.1 | slg:SLGD_00784 | 50S ribosomal protein L5 | 20232 | 8837,80476 | 5668,27012 | 1,55917142 |
| WP_002479199.1 | slg:SLGD_02551 | DNA gyrase subunit A | 99890 | 9258,06311 | 4136,9162 | 2,2379141 |
| WP_002478237.1 | slg:SLGD_01547 | hypothetical protein | 11612 | 1464,10559 | 1047,13407 | 1,39820262 |
| WP_012990787.1 | slg:SLGD_00950 | anti-sigma B factor RsbW | 17796 | 747,588726 | 1543,48049 | 2,06461178 |
| WP_002458757.1 | slg:SLGD_01107 | glucosamine-6-phosphate isomerase | 22606 | 33554,2979 | 22525,5863 | 1,48960819 |
| WP_002459324.1 | slg:SLGD_01682 | 50S ribosomal protein L28 | 6967 | 4618,40326 | 1630,8219 | 2,83194826 |
| WP_002460063.1 | slg:SLGD_00779 | 50S ribosomal protein L16 | 16303 | 764,291486 | 436,418243 | 1,75128217 |
| WP_002478172.1 | slg:SLGD_01434 | HU family DNA-binding protein | 9636 | 107221,74 | 801403,635 | 7,47426441 |
| WP_037557155.1 | slg:SLGD_01619 | ribonuclease J | 61281 | 25116,9034 | 49958,3627 | 1,98903352 |
| WP_002478342.1 | slg:SLGD_01739 | DNA polymerase/3'-5' exonuclease PolX | 64809 | 2030,44094 | 1423,06451 | 1,42680878 |
| WP_002459942.1 | slg:SLGD_02353 | transcription termination/antitermination protein NusG | 20613 | 7156,07284 | 14728,6321 | 2,05820042 |
| WP_002458895.1 | slg:SLGD_01251 | 50S ribosomal protein L20 | 13709 | 1720,2505 | 645,172274 | 2,66634288 |
| WP_002459854.1 | slg:SLGD_02209 | transcriptional regulator | 14398 | 118361,622 | 35193,8452 | 3,36313412 |
| WP_002459691.1 | slg:SLGD_02050 | phosphopyruvate hydratase | 47403 | 438039,304 | 284882,312 | 1,53761496 |
| WP_002478940.1 | slg:SLGD_00876 | DNA starvation/stationary phase protection protein | 16876 | 131670,426 | 174305,993 | 1,32380519 |
| WP_002461247.1 | slg:SLGD_00904 | type B 50S ribosomal protein L31 | 9787 | 281,274384 | 1455,02651 | 5,17297912 |
| WP_002478986.1 | slg:SLGD_00301 | hypothetical protein | 73364 | 48397,8198 | 77101,6737 | 1,59308155 |
| WP_002459857.1 | slg:SLGD_02212 | hypothetical protein | 18569 | 4103058,25 | 2434684,13 | 1,6852528 |
| WP_002478811.1 | slg:SLGD_00741 | ferrichrome ABC transporter substrate-binding protein | 33976 | 13753,7722 | 7935,80652 | 1,73312847 |
| WP_002478415.1 | slg:SLGD_01862 | NAD kinase | 30603 | 5273,33725 | 12400,2616 | 2,35150172 |
| WP_002478394.1 | slg:SLGD_01827 | 1,4-dihydroxy-2-naphthoyl-CoA synthase | 30319 | 3475,11326 | 1871,3662 | 1,85699264 |
| WP_002460496.1 | slg:SLGD_00435 | L-glutamate gamma-semialdehyde dehydrogenase | 56679 | 1465,99426 | 4095,75495 | 2,79384106 |
| WP_002448459.1 | slg:SLGD_00783 | 50S ribosomal protein L24 [Bacilli] | 11513 | 20876,5605 | 6092,50802 | 3,42659549 |
| WP_002458855.1 | slg:SLGD_01211 | 30S ribosomal protein S4 | 23149 | 7946,06035 | 5304,94847 | 1,49785816 |
| WP_002477933.1 | slg:SLGD_01007 | aldehyde dehydrogenase | 51705 | 267,649491 | 5662,93071 | 21,1580104 |
| WP_002458920.1 | slg:SLGD_01276 | 50S ribosomal protein L27 | 10321 | 15728,638 | 9024,29782 | 1,74292098 |
| WP_002478941.1 | slg:SLGD_00878 | deoxyribose-phosphate aldolase | 23281 | 6136,01232 | 2372,62147 | 2,58617415 |
| WP_002478109.1 | slg:SLGD_01334 | nucleotide exchange factor GrpE | 23730 | 994,125173 | 108,85552 | 9,13251959 |
| WP_002459379.1 | slg:SLGD_01737 | thioredoxin | 11488 | 2910,7634 | 1018,63097 | 2,85752495 |
| WP_002478123.1 | slg:SLGD_01358 | deoxyribonuclease IV | 32877 | 14618,3152 | 28259,1456 | 1,93313287 |
| WP_012990855.1 | slg:SLGD_01556 | transketolase | 72804 | 45483,1925 | 25357,3054 | 1,79369187 |
| WP_002479013.1 | slg:SLGD_00263 | N-acetylglucosamine-6-phosphate deacetylase | 42150 | 1873,15649 | 691,005069 | 2,71077098 |
| WP_002460725.1 | slg:SLGD_00246 | ornithine decarboxylase | 82770 | 369161,833 | 721059,109 | 1,95323309 |
| WP_002478410.1 | slg:SLGD_01853 | hypothetical protein | 19314 | 10085,6276 | 5172,01819 | 1,95003715 |
| WP_002478917.1 | slg:SLGD_00837 | Asp23/Gls24 family envelope stress response protein | 18958 | 40965,0359 | 19536,6409 | 2,09683108 |
| WP_002460303.1 | slg:SLGD_00581 | amino acid ABC transporter substrate-binding protein | 28676 | 2463,5881 | 1622,16263 | 1,51870599 |
| WP_002459095.1 | slg:SLGD_01451 | asparagine--tRNA ligase | 49267 | 1390,83643 | 512,514888 | 2,71374834 |
| WP_002478996.1 | slg:SLGD_00289 | SMP-30/gluconolactonase/LRE family protein | 35897 | 241,600781 | 1522,57652 | 6,30203474 |
| WP_002478682.1 | slg:SLGD_00562 | NAD(P)-dependent oxidoreductase | 31228 | 13756,7179 | 6215,80884 | 2,21318226 |
| WP_002459033.1 | slg:SLGD_01389 | Asp23/Gls24 family envelope stress response protein | 13177 | 1841,0539 | 678,293547 | 2,71424358 |
| WP_002478655.1 | slg:SLGD_00522 | M42 family peptidase | 39205 | 12218,3152 | 17229,5467 | 1,41014096 |
| WP_002460367.1 | slg:SLGD_00528 | phosphate/phosphite/phosphonate ABC transporter substrate-binding protein | 35060 | 11560,9353 | 6157,00147 | 1,87768922 |
| WP_002478153.1 | slg:SLGD_01408 | glucose-6-phosphate dehydrogenase | 56947 | 37575,3611 | 76076,1509 | 2,02462861 |
| WP_002478684.1 | slg:SLGD_00566 | hypothetical protein | 35406 | 21069,4895 | 12127,4213 | 1,73734292 |
| WP_002460061.1 | slg:SLGD_00782 | 50S ribosomal protein L14 | 13128 | 39658,9296 | 20387,8195 | 1,94522664 |
| WP_002460067.1 | slg:SLGD_00775 | 50S ribosomal protein L2 | 30237 | 8587,4127 | 13541,1353 | 1,57685856 |
| WP_002459941.1 | slg:SLGD_02352 | 50S ribosomal protein L11 | 14895 | 10999,9057 | 3821,02955 | 2,8787806 |
| WP_002478269.1 | slg:SLGD_01615 | insulinase family protein | 50067 | 4194,35072 | 1281,04411 | 3,27416574 |
| WP_012990738.1 | slg:SLGD_00578 | phosphoglycerate mutase | 26591 | 64702,2721 | 113348,699 | 1,75185036 |
| WP_002460813.1 | slg:SLGD_00178 | 3-oxoacyl-ACP reductase | 28023 | 3617,31013 | 1289,92007 | 2,80429013 |
| WP_002478128.1 | slg:SLGD_01368 | glucokinase | 34923 | 418,80226 | 1716,42219 | 4,09840717 |
| WP_002461102.1 | slg:SLGD_02492 | single-stranded DNA-binding protein | 18736 | 36886,2184 | 21703,4777 | 1,69955336 |
| WP_002478163.1 | slg:SLGD_01419 | rRNA pseudouridine synthase | 27834 | 1274,12763 | 821,440349 | 1,55108966 |
| WP_002477932.1 | slg:SLGD_01006 | hypothetical protein | 38589 | 4799,37587 | 8544,99872 | 1,78043957 |
| WP_002478004.1 | slg:SLGD_01155 | transaldolase | 25698 | 18770,9697 | 31230,9432 | 1,66378955 |
| WP_002478110.1 | slg:SLGD_01335 | molecular chaperone DnaK | 66328 | 103310,85 | 61909,0758 | 1,66875128 |
| WP_002478221.1 |  | PDZ domain-containing protein | 54326 | 1004,09488 | 381,826335 | 2,62971614 |
| WP_002459323.1 | slg:SLGD_01681 | Asp23/Gls24 family envelope stress response protein | 13477 | 4093,39162 | 2179,6981 | 1,87796265 |
| WP_002459533.1 | slg:SLGD_01890 | FAA hydrolase family protein | 32986 | 41222,969 | 17045,2033 | 2,41844983 |
| WP_002478087.1 | slg:SLGD_01291 | aspartate--tRNA ligase | 66641 | 2429,67951 | 1106,69217 | 2,19544293 |
| WP_002460777.1 | slg:SLGD_00206 | hypothetical protein | 18961 | 3764840,87 | 1802607,91 | 2,08855228 |
| WP_002459255.1 | slg:SLGD_01605 | 2-oxoacid:acceptor oxidoreductase subunit alpha | 64577 | 2578,28057 | 934,449689 | 2,75914327 |
| WP_002459424.1 | slg:SLGD_01781 | alpha-ketoacid dehydrogenase subunit beta | 35296 | 361847,982 | 183694,112 | 1,96983985 |
| WP_012990944.1 | slg:SLGD_02129 | PTS fructose transporter subunit IIC | 69090 | 5709,01758 | 2823,33927 | 2,02207989 |
| WP_002461472.1 | slg:SLGD_02407 | 50S ribosomal protein L25 | 23665 | 52642,9645 | 20252,4609 | 2,59933668 |
| WP_002461018.1 | slg:SLGD_02562 | adenylosuccinate synthase | 47434 | 1156,18067 | 332,027952 | 3,4821787 |
| WP_002460068.1 | slg:SLGD_00774 | 50S ribosomal protein L23 | 10625 | 5229,58672 | 1333,61737 | 3,92135468 |
| WP_002479252.1 | slg:SLGD_02472 | IMP dehydrogenase | 52719 | 126077,232 | 35347,7658 | 3,56676663 |
| WP_002460510.1 | slg:SLGD_00424 | transglycosylase | 24142 | 692462,192 | 143212,1 | 4,83522127 |
| WP_002477958.1 | slg:SLGD_01039 | CoA-disulfide reductase | 50132 | 14018,4241 | 2386,27794 | 5,87459821 |
| WP_002478071.1 | slg:SLGD_01265 | valine--tRNA ligase | 101401 | 618,99668 | 15,4374052 | 40,0971972 |
| WP_002478117.1 | slg:SLGD_01350 | glycine--tRNA ligase | 53551 | 1265,35546 | 384,717885 | 3,28904765 |
| WP_002460046.1 | slg:SLGD_00799 | 50S ribosomal protein L17 | 13682 | 453071,652 | 73627,0864 | 6,15360018 |
| WP_002460049.1 | slg:SLGD_00796 | 30S ribosomal protein S13 | 13737 | 16722,7625 | 5985,26826 | 2,79398712 |
| WP_002461066.1 | slg:SLGD_02526 | signal peptidase I | 21985 | 2697,11652 | 542,602619 | 4,9707031 |
| WP_002458974.1 | slg:SLGD_01329 | 30S ribosomal protein S20 | 8946 | 14364,9418 | 1309,99835 | 10,9656183 |
| WP_002460040.1 | slg:SLGD_00805 | 30S ribosomal protein S9 | 14678 | 6387,06674 | 1549,69109 | 4,12150962 |
| WP_002460054.1 | slg:SLGD_00789 | 30S ribosomal protein S5 | 17715 | 33322,4541 | 9399,22545 | 3,54523405 |
| WP_002459075.1 | slg:SLGD_01431 | 30S ribosomal protein S1 | 43114 | 698,275521 | 60,2841822 | 11,5830637 |
| WP_002460058.1 | slg:SLGD_00786 | 30S ribosomal protein S8 | 14812 | 22587,1068 | 5101,90262 | 4,42719286 |
| WP_002459256.1 | slg:SLGD_01606 | TIGR00282 family metallophosphoesterase | 29456 | 879,246399 | 212,441725 | 4,13876512 |
| WP_002478985.1 | slg:SLGD_00302 | CHAP domain-containing protein | 73923 | 66476,9327 | 20217,1464 | 3,28814618 |
| WP_002479363.1 | slg:SLGD_02116 | LTA synthase family protein | 74826 | 1503790,34 | 463015,107 | 3,24782134 |
| WP_014533190.1 | slg:SLGD_00044 | tandem-type lipoprotein | 30500 | 3520,62294 | 476,590485 | 7,38710286 |
| WP_002460048.1 | slg:SLGD_00798 | DNA-directed RNA polymerase subunit alpha | 34990 | 37287,1896 | 10917,8031 | 3,4152649 |
| WP_002478964.1 | slg:SLGD_00917 | F0F1 ATP synthase subunit delta | 20431 | 6805,91391 | 1625,23122 | 4,18765885 |
| WP_002459361.1 | slg:SLGD_01718 | cell division protein FtsA | 52417 | 1609,40313 | 364,686031 | 4,41311976 |
| WP_002458789.1 | slg:SLGD_01148 | phosphoenolpyruvate carboxykinase (ATP) | 59305 | 1954,74767 | 413,936147 | 4,72234108 |
| WP_002478454.1 | slg:SLGD_01917 | leucyl aminopeptidase family protein | 53996 | 1093,972 | 238,125753 | 4,59409361 |
| WP_002459500.1 | slg:SLGD_01858 | enoyl-[acyl-carrier-protein] reductase FabI | 28028 | 22946,222 | 2071,35157 | 11,0778982 |
| WP_002458998.1 | slg:SLGD_01354 | RNA polymerase sigma factor RpoD | 42330 | 1497,39625 | 564,247105 | 2,65379518 |
| WP_002492333.1 | slg:SLGD_01438 | nucleoside-diphosphate kinase | 16393 | 2229,56014 | 265,400297 | 8,4007447 |
| WP_001137495.1 | slg:SLGD_02343 | 30S ribosomal protein S7 [Bacilli] | 17783 | 14338,2891 | 1475,37289 | 9,71841704 |
| WP_002461476.1 | slg:SLGD_02410 | septation protein SpoVG | 11414 | 13911,0991 | 2555,86281 | 5,44281919 |
| WP_012990671.1 | slg:SLGD_00171 | formate C-acetyltransferase | 84838 | 48411,2833 | 11092,3029 | 4,3644033 |
| WP_002479141.1 | slg:SLGD_00090 | YSIRK signal domain/LPXTG anchor domain surface protein | 76829 | 18346,5429 | 8631,7038 | 2,12548337 |
| WP_002479219.1 | slg:SLGD_02520 | hypothetical protein | 14658 | 876603,079 | 22532,0981 | 38,9046361 |
| WP_002459774.1 | slg:SLGD_02133 | Cys-tRNA(Pro) deacylase | 18051 | 9236,71288 | 3315,65304 | 2,78578994 |
| WP_002478366.1 | slg:SLGD_01780 | 2-oxo acid dehydrogenase subunit E2 | 46852 | 42886,6529 | 10920,6671 | 3,92710927 |
| WP_002461237.1 | slg:SLGD_00912 | uracil phosphoribosyltransferase | 23071 | 1106,72193 | 45,5734093 | 24,2843787 |
| WP_002479036.1 | slg:SLGD_00231 | alpha-ketoacid dehydrogenase subunit beta | 36887 | 7508,33537 | 427,790165 | 17,5514446 |
| WP_002461385.1 | slg:SLGD_01008 | manganese-dependent inorganic pyrophosphatase | 34201 | 18387,4239 | 5330,88575 | 3,4492249 |
| WP_002459289.1 | slg:SLGD_01639 | 30S ribosomal protein S2 | 29719 | 51998,7858 | 20892,7099 | 2,48884831 |
| WP_012990817.1 | slg:SLGD_01220 | universal stress protein | 18482 | 37943,5196 | 13964,1892 | 2,71720178 |
| WP_002479342.1 | slg:SLGD_02077 | ribosomal subunit interface protein | 21940 | 13556,1305 | 3027,60092 | 4,47751567 |
| WP_002459304.1 | slg:SLGD_01655 | 50S ribosomal protein L19 | 13371 | 8592,92817 | 2955,15924 | 2,90777162 |
| WP_002459425.1 | slg:SLGD_01782 | pyruvate dehydrogenase (acetyl-transferring) E1 component subunit alpha | 41588 | 441999,64 | 81441,5961 | 5,42719767 |
| WP_002458938.1 | slg:SLGD_01294 | Rrf2 family transcriptional regulator | 15532 | 7650,65419 | 3380,90652 | 2,26290024 |
| WP_002478833.1 | slg:SLGD_00793 | adenylate kinase | 24106 | 7187,53503 | 520,052993 | 13,8207743 |
| WP_002460442.1 | slg:SLGD_00476 | lactate dehydrogenase | 36218 | 60731,5576 | 22704,9356 | 2,67481744 |
| WP_002479438.1 | slg:SLGD_02309 | bifunctional hydroxymethylpyrimidine kinase/phosphomethylpyrimidine kinase | 29557 | 3800,84071 | 584,129789 | 6,5068428 |
| WP_002461120.1 | slg:SLGD_02475 | hypothetical protein | 15426 | 12040,5492 | 1123,35447 | 10,7183881 |
| WP_002460100.1 | slg:SLGD_00746 | molybdate ABC transporter substrate-binding protein | 28652 | 52438,6243 | 25807,1828 | 2,03193912 |
| WP_002458844.1 | slg:SLGD_01202 | tyrosine--tRNA ligase | 47492 | 2362,62026 | 545,44878 | 4,33151625 |
| WP_002479495.1 | slg:SLGD_02395 | cysteine synthase A | 32929 | 8439,75392 | 922,178336 | 9,15197591 |
| WP_002459548.1 | slg:SLGD_01905 | peptidyl-prolyl cis-trans isomerase | 21654 | 1704,71381 | 308,515958 | 5,5255288 |
| WP_002460041.1 | slg:SLGD_00804 | 50S ribosomal protein L13 | 16267 | 29634,9511 | 3964,23436 | 7,47558 |
| WP_002479403.1 | slg:SLGD_02189 | formate dehydrogenase | 37754 | 13987,6857 | 2751,28512 | 5,08405529 |
| WP_012990815.1 | slg:SLGD_01203 | PDZ domain-containing protein | 43608 | 1203,19321 | 206,624853 | 5,82308077 |
| WP_002459457.1 | slg:SLGD_01813 | DUF5011 domain-containing protein | 11460 | 33831,5153 | 1446,43851 | 23,3895289 |
| WP_002458918.1 | slg:SLGD_01274 | 50S ribosomal protein L21 | 11286 | 10306,5702 | 1105,4527 | 9,3233932 |
| WP_002459623.1 | slg:SLGD_01980 | Fe-S cluster assembly protein SufB | 52514 | 2987,48245 | 794,426153 | 3,760554 |
| WP_002458835.1 | slg:SLGD_01194 | catabolite control protein A | 36387 | 7987,68907 | 650,302408 | 12,2830378 |
| WP_002459699.1 | slg:SLGD_02058 | ATP-dependent Clp endopeptidase, proteolytic subunit ClpP | 21413 | 10769,1327 | 1701,20217 | 6,33030745 |
| WP_002477971.1 | slg:SLGD_01065 | glutamate-1-semialdehyde 2,1-aminomutase | 46963 | 3746,61405 | 1246,03643 | 3,00682546 |
| WP_002460439.1 | slg:SLGD_00479 | aldehyde dehydrogenase | 54175 | 5331,25851 | 937,074975 | 5,68925503 |
| WP_002477986.1 | slg:SLGD_01122 | peptidyl-prolyl cis-trans isomerase | 36111 | 112601,216 | 5417,67855 | 20,7840341 |
| WP_012990866.1 | slg:SLGD_01649 | succinate--CoA ligase subunit alpha | 31476 | 2130,10051 | 56,4666883 | 37,7231352 |
| WP_002478501.1 | slg:SLGD_00327 | KR domain-containing protein | 38482 | 866,856518 | 78,9702005 | 10,9770079 |
| WP_002478262.1 | slg:SLGD_01592 | glycerol kinase | 56114 | 133189,317 | 3452,66502 | 38,5758004 |
| WP_002478068.1 | slg:SLGD_01261 | porphobilinogen synthase | 36346 | 1564,313 | 195,49614 | 8,00175904 |
| WP_012990779.1 | slg:SLGD_00840 | iron citrate ABC transporter substrate-binding protein | 36988 | 28433,3352 | 6877,43481 | 4,13429367 |
| WP_002478832.1 | slg:SLGD_00787 | 50S ribosomal protein L6 | 19556 | 74030,3766 | 15843,1624 | 4,672702 |
| WP_002458875.1 | slg:SLGD_01231 | 6-phosphofructokinase | 34703 | 6125,60144 | 2544,59588 | 2,40729835 |
| WP_002459307.1 | slg:SLGD_01658 | 30S ribosomal protein S16 | 10342 | 36099,8623 | 1144,23011 | 31,5494775 |
| WP_002478929.1 | slg:SLGD_00864 | phosphoglucosamine mutase | 48826 | 3664,18921 | 493,666836 | 7,42239288 |
| WP_002461020.1 | slg:SLGD_02560 | 50S ribosomal protein L9 | 16296 | 57636,2315 | 6935,35074 | 8,31049988 |
| WP_002478135.1 | slg:SLGD_01380 | glycine dehydrogenase subunit 2 | 54792 | 1500,63709 | 39,3703675 | 38,1159025 |
| WP_002478385.1 | slg:SLGD_01812 | nitric oxide dioxygenase | 43082 | 4088,18965 | 146,329934 | 27,9381638 |
| WP_002460056.1 | slg:SLGD_00788 | 50S ribosomal protein L18 | 13206 | 54190,3254 | 2923,3295 | 18,5371938 |
| WP_002461245.1 | slg:SLGD_00906 | peptide chain release factor 1 | 40473 | 6630,00657 | 1329,00482 | 4,98870018 |
| WP_002461359.1 | slg:SLGD_01027 | Asp-tRNA(Asn)/Glu-tRNA(Gln) amidotransferase subunit GatA | 52847 | 5534,15718 | 740,116369 | 7,47741492 |
| WP_002459064.1 | slg:SLGD_01420 | DNA-binding response regulator | 28083 | 5518,44866 | 1354,06292 | 4,07547432 |
| WP_002478831.1 | slg:SLGD_00776 | 30S ribosomal protein S19 | 10581 | 58160,4407 | 6106,87724 | 9,52376123 |
| WP_002459561.1 | slg:SLGD_01918 | NAD(P)/FAD-dependent oxidoreductase | 44284 | 15199,2456 | 1623,54605 | 9,36175825 |
| WP_002479465.1 | slg:SLGD_02351 | 50S ribosomal protein L1 | 24850 | 41212,499 | 14836,7825 | 2,77772482 |
| WP_002477948.1 | slg:SLGD_01028 | Asp-tRNA(Asn)/Glu-tRNA(Gln) amidotransferase subunit GatB | 53595 | 7467,74826 | 331,399151 | 22,5339994 |
| WP_037557156.1 | slg:SLGD_01627 | DUF448 domain-containing protein | 9979 | 4687,23237 | 154,530014 | 30,3321811 |
| WP_002478423.1 | slg:SLGD_01875 | peptide ABC transporter substrate-binding protein | 61569 | 1388,7965 | 88,5521517 | 15,6833738 |
| WP_002478497.1 | slg:SLGD_00321 | azoreductase | 23008 | 655,73523 | 38,4102811 | 17,0718675 |
| WP_002459693.1 | slg:SLGD_02052 | triose-phosphate isomerase | 27424 | 61208,2867 | 9017,94647 | 6,78738635 |
| WP_002459694.1 | slg:SLGD_02053 | phosphoglycerate kinase | 42201 | 84093,1189 | 5286,93608 | 15,9058323 |
| WP_002459931.1 | slg:SLGD_02341 | elongation factor Tu | 43083 | 19653,919 | 4240,34817 | 4,63497765 |
| WP_002459799.1 | slg:SLGD_02156 | YebC/PmpR family DNA-binding transcriptional regulator | 26356 | 11213,2258 | 377,369581 | 29,7141751 |
| WP_002478136.1 | slg:SLGD_01384 | hypothetical protein | 22006 | 9339,14761 | 200,94876 | 46,4752687 |
| WP_002477926.1 | slg:SLGD_00992 | chaperonin GroEL | 57587 | 58328,1374 | 20292,0393 | 2,87443448 |
| WP_002479459.1 | slg:SLGD_02339 | glycine C-acetyltransferase | 42730 | 5907,7777 | 702,008079 | 8,4155409 |
| WP_002478326.1 | slg:SLGD_01710 | isoleucine--tRNA ligase | 104923 | 6946,28322 | 283,522882 | 24,4999034 |
| WP_002459883.1 | slg:SLGD_02297 | phosphate acetyltransferase | 34794 | 17337,3675 | 5124,12433 | 3,38347909 |
| WP_002479326.1 | slg:SLGD_02051 | 2,3-bisphosphoglycerate-independent phosphoglycerate mutase | 56594 | 13990,345 | 3071,33197 | 4,55513932 |
| WP_002459921.1 | slg:SLGD_02330 | bifunctional threonine ammonia-lyase/L-serine ammonia-lyase TdcB | 37080 | 87147,4801 | 17005,128 | 5,12477647 |
| WP_002459160.1 | slg:SLGD_01515 | PTS glucose transporter subunit IIA | 17906 | 15361,5525 | 2911,64845 | 5,27589535 |
| WP_002479422.1 | slg:SLGD_02225 | alcohol dehydrogenase AdhP | 35969 | 23674,0247 | 1686,54373 | 14,0370061 |
| WP_002492254.1 | slg:SLGD_00738 | ATP-dependent Clp protease ATP-binding subunit | 78182 | 1874,4643 | 616,519924 | 3,04039533 |
| WP_002478624.1 | slg:SLGD_00478 | YSIRK signal domain/LPXTG anchor domain surface protein | 30578 | 23699,2687 | 697,42673 | 33,9810158 |
| WP_002460169.1 | slg:SLGD_00686 | HlyD family secretion protein | 22686 | 3006,84821 | 309,787699 | 9,70615753 |
| WP_002478302.1 | slg:SLGD_01677 | phosphate acyltransferase PlsX | 35336 | 2707,82459 | 228,383656 | 11,8564727 |
| WP_002478960.1 | slg:SLGD_00911 | serine hydroxymethyltransferase | 44956 | 3212,21347 | 488,40756 | 6,57691186 |
| WP_002459422.1 | slg:SLGD_01779 | dihydrolipoyl dehydrogenase | 49606 | 95329,4863 | 18531,6118 | 5,14415514 |
| WP_002458986.1 | slg:SLGD_01342 | UPF0365 family protein | 35061 | 3267,27252 | 282,420051 | 11,5688405 |
| WP_002458891.1 | slg:SLGD_01247 | threonine--tRNA ligase | 74875 | 5319,55081 | 374,378391 | 14,2090221 |
| WP_002461270.1 | slg:SLGD_00880 | phosphopentomutase | 43996 | 22064,6172 | 1770,97382 | 12,4590307 |
| WP_012990956.1 | slg:SLGD_02220 | arginine--tRNA ligase | 62792 | 2092,7891 | 36,2952898 | 57,6600743 |
| WP_002479104.1 | slg:SLGD_00139 | threonine--tRNA ligase | 73876 | 56176,0772 | 6069,20591 | 9,25591882 |
| WP_002479043.1 | slg:SLGD_00224 | flavocytochrome c | 111380 | 11190,9546 | 816,72514 | 13,7022287 |
| WP_002478063.1 | slg:SLGD_01254 | trigger factor | 48915 | 13458,4573 | 346,731542 | 38,8152091 |
| WP_002479198.1 | slg:SLGD_02554 | serine--tRNA ligase | 48752 | 71490,0456 | 4699,43438 | 15,2124787 |
| WP_002458832.1 | slg:SLGD_01191 | DUF948 domain-containing protein | 17264 | 9072,30613 | 407,659908 | 22,2545949 |
| WP_002459058.1 | slg:SLGD_01414 | transcriptional repressor | 17708 | 1930,96342 | 266,102454 | 7,25646605 |
| WP_002478950.1 | slg:SLGD_00897 | CTP synthase | 59793 | 2531,72889 | 100,673108 | 25,1480157 |
| WP_002478386.1 | slg:SLGD_01814 | cytochrome aa3 quinol oxidase subunit II | 42203 | 12222,2917 | 984,332548 | 12,4168319 |
| WP_002478253.1 | slg:SLGD_01579 | ABC transporter ATP-binding protein | 33288 | 2758,9483 | 57,5826829 | 47,9128128 |
| WP_002479499.1 | slg:SLGD_02390 | lysine--tRNA ligase | 56969 | 7188,773 | 823,547986 | 8,72902748 |
| WP_012990814.1 | slg:SLGD_01200 | formate--tetrahydrofolate ligase | 59822 | 14192,5853 | 968,977638 | 14,6469689 |
| WP_002459960.1 | slg:SLGD_02370 | pyridoxal 5'-phosphate synthase lyase subunit PdxS | 31897 | 17112,1341 | 2457,60084 | 6,96294281 |
| WP_002459582.1 | slg:SLGD_01941 | D-alanine--poly(phosphoribitol) ligase subunit 1 | 54358 | 4779,36637 | 366,397024 | 13,0442281 |
| WP_002460064.1 | slg:SLGD_00778 | 30S ribosomal protein S3 | 24172 | 3032,84884 | 333,571105 | 9,092061 |
| WP_002478556.1 | slg:SLGD_00388 | fructose bisphosphate aldolase | 33029 | 219062,311 | 43954,4272 | 4,98385089 |
| WP_002478550.1 | slg:SLGD_00379 | malate dehydrogenase (quinone) | 56317 | 7329,71787 | 2058,09079 | 3,5614162 |
| WP_002478061.1 | slg:SLGD_01252 | NUDIX domain-containing protein | 23099 | 2091,19029 | 188,611445 | 11,0872926 |
| WP_002478388.1 | slg:SLGD_01820 | LytR family transcriptional regulator | 45180 | 117916,353 | 15001,0303 | 7,86055032 |
| WP_002461113.1 | slg:SLGD_02482 | alkyl hydroperoxide reductase subunit F | 54861 | 5991,35617 | 313,443672 | 19,1146184 |
| WP_002479468.1 | slg:SLGD_02359 | cysteine--tRNA ligase | 53755 | 5309,15633 | 234,994024 | 22,5927291 |
| WP_002459695.1 | slg:SLGD_02054 | type I glyceraldehyde-3-phosphate dehydrogenase | 36172 | 433876,755 | 47854,392 | 9,06660261 |
| WP_002478951.1 | slg:SLGD_00899 | fructose-1,6-bisphosphate aldolase, class II | 30723 | 54883,7467 | 11197,1916 | 4,90156359 |
| WP_002479029.1 | slg:SLGD_00239 | mannose-6-phosphate isomerase, class I | 35074 | 1297,32168 | 69,6687675 | 18,6212808 |
| WP_002459360.1 | slg:SLGD_01717 | cell division protein FtsZ | 41227 | 31762,3519 | 597,924723 | 53,1209878 |
| WP_002492284.1 | slg:SLGD_00724 | 2-hydroxyacid dehydrogenase | 34791 | 5875,93754 | 313,015337 | 18,7720436 |
| WP_002478930.1 | slg:SLGD_00865 | glutamine--fructose-6-phosphate transaminase (isomerizing) | 65844 | 7764,89185 | 860,470974 | 9,02400207 |
| WP_002479461.1 | slg:SLGD_02342 | elongation factor G | 76611 | 32655,9576 | 2736,24487 | 11,9345889 |
| WP_002458953.1 | slg:SLGD_01309 | transcription elongation factor GreA | 17810 | 7319,01818 | 225,174988 | 32,5036908 |
| WP_002459099.1 | slg:SLGD_01455 | penicillin-binding protein | 80977 | 6862,30716 | 625,881193 | 10,9642329 |
| WP_002479067.1 | slg:SLGD_00191 | hypothetical protein | 35472 | 23756,056 | 726,884685 | 32,6820147 |
| WP_002459939.1 | slg:SLGD_02350 | 50S ribosomal protein L10 | 17708 | 28841,7718 | 2194,30304 | 13,1439328 |
| WP_002460126.1 | slg:SLGD_00719 | LytR family transcriptional regulator | 35041 | 1267,70646 | 5681,86499 | 4,48200364 |
| WP_002478368.1 | slg:SLGD_01786 | RNase J family beta-CASP ribonuclease | 61914 | 11990,9845 | 48409,0294 | 4,03711883 |
| WP_002479246.1 | slg:SLGD_02481 | peroxiredoxin | 21046 | 146527,259 | 342630,949 | 2,33834272 |
| WP_002478793.1 | slg:SLGD_00717 | HTH domain-containing protein | 26164 | 300,44062 | 860,791198 | 2,86509593 |
| WP_002478443.1 | slg:SLGD_01903 | NADH-dependent flavin oxidoreductase | 42186 | 1681,86883 | 7491,651 | 4,45436104 |
| WP_002479452.1 | slg:SLGD_02329 | alanine dehydrogenase | 39627 | 241269,807 | 463262,674 | 1,92010214 |
| WP_002478761.1 | slg:SLGD_00670 | hypothetical protein | 23888 | 6527,34832 | 19925,2493 | 3,05257944 |
| WP_002478600.1 | slg:SLGD_00451 | LysR family transcriptional regulator | 33330 | 2863,46321 | 7962,29511 | 2,78065214 |
| WP_002459294.1 | slg:SLGD_01644 | FADH(2)-oxidizing methylenetetrahydrofolate--tRNA-(uracil(54)-C(5))-methyltransferase TrmFO | 48246 | 213,270073 | 517,237762 | 2,42527118 |
| WP_002478931.1 | slg:SLGD_00866 | Cof-type HAD-IIB family hydrolase | 31987 | 1697,44947 | 4149,00775 | 2,44425995 |
| WP_012990793.1 | slg:SLGD_00993 | aspartate aminotransferase | 48564 | 8041,52348 | 16033,3641 | 1,99382171 |
| WP_002460471.1 | slg:SLGD_00449 | CHAP domain-containing protein | 16701 | 12402,6825 | 257182,225 | 20,7360162 |
| WP_002477963.1 | slg:SLGD_01050 | aminopeptidase | 46350 | 3759,55433 | 15946,164 | 4,2415038 |
| WP_002458866.1 | slg:SLGD_01222 | aminopeptidase P family protein | 39712 | 7751,64843 | 22328,3857 | 2,88046935 |
| WP_002478419.1 | slg:SLGD_01868 | oligoendopeptidase F | 69958 | 18487,5696 | 57280,371 | 3,09831808 |
| WP_002460942.1 | slg:SLGD_00048 | L-lactate dehydrogenase | 34631 | 104124,333 | 201321,435 | 1,93347154 |
| WP_002459545.1 | slg:SLGD_01902 | Glu/Leu/Phe/Val dehydrogenase | 45802 | 12584,2492 | 30638,8394 | 2,43469744 |
| WP_002459223.1 | slg:SLGD_01574 | thermonuclease | 20653 | 1568,39018 | 8089,7317 | 5,15798415 |
| WP_002478367.1 | slg:SLGD_01784 | peptide deformylase | 20519 | 3964,33641 | 14807,8295 | 3,73526059 |
| WP_002478121.1 | slg:SLGD_01356 | Nif3-like dinuclear metal center hexameric protein | 41350 | 896,895362 | 2572,78266 | 2,86854272 |
| WP_002479472.1 | slg:SLGD_02369 | pyridoxal 5'-phosphate synthase glutaminase subunit PdxT | 20450 | 655,737986 | 18594,1829 | 28,3561168 |
| WP_002459157.1 | slg:SLGD_01512 | N-acetyltransferase | 19416 | 5774,23375 | 11553,5724 | 2,00088409 |
| WP_002479056.1 | slg:SLGD_00209 | polyisoprenoid-binding protein | 19101 | 1645,84714 | 11847,0315 | 7,19813599 |
| WP_002478018.1 | slg:SLGD_01185 | M42 family peptidase | 39831 | 2316,82134 | 13655,2362 | 5,89395302 |
| WP_002478034.1 | slg:SLGD_01210 | glycerophosphodiester phosphodiesterase | 28597 | 2640,86554 | 8155,5895 | 3,08822595 |
| WP_012990650.1 |  | hypothetical protein, partial | 11141 | 757157,007 | 2452215,88 | 3,23871517 |
| WP_012990705.1 | slg:SLGD_00398 | LysR family transcriptional regulator | 33844 | 2161,38623 | 10623,4671 | 4,91511742 |
| WP_002460628.1 | slg:SLGD_00330 | antibiotic acetyltransferase | 23106 | 184,627117 | 2429,11059 | 13,1568462 |
| WP_012990863.1 | slg:SLGD_01633 | methicillin resistance protein | 76481 | 31415,4163 | 96385,7661 | 3,06810405 |
| WP_012990972.1 | slg:SLGD_02246 | phage major tail protein, TP901-1 family | 20765 | 384,958028 | 9160,23741 | 23,7954186 |
| WP_002478587.1 | slg:SLGD_00432 | sugar O-acetyltransferase | 21034 | 2078,66113 | 10610,2589 | 5,10437162 |
| WP_002478707.1 | slg:SLGD_00596 | uroporphyrinogen-III C-methyltransferase | 34102 | 178,580983 | 3499,57948 | 19,5965965 |
| WP_012990967.1 | slg:SLGD_02241 | hypothetical protein | 41559 | 399,298766 | 1949,32441 | 4,88186936 |
| WP_002477992.1 | slg:SLGD_01128 | hypothetical protein | 19296 | 4935,35436 | 37139,7841 | 7,52525175 |
| WP_002459884.1 | slg:SLGD_02298 | heme-dependent peroxidase | 29288 | 21136,6197 | 49666,2016 | 2,34977032 |
| WP_012990978.1 | slg:SLGD_02252 | hypothetical protein | 32710 | 33189,9717 | 242308,616 | 7,3006575 |
| WP_002478255.1 | slg:SLGD_01582 | type I glutamate--ammonia ligase | 50931 | 24642,8542 | 183597,893 | 7,45035019 |
| WP_014533295.1 | slg:SLGD_01351 | transcriptional regulator | 23691 | 706,48599 | 2856,28483 | 4,04294617 |
| WP_002478817.1 | slg:SLGD_00754 | molybdenum cofactor biosynthesis protein MoaE | 17348 | 96,1147132 | 449,605197 | 4,67779783 |
| WP_002479290.1 | slg:SLGD_01965 | cystatin-like fold lipoprotein | 15126 | 137,71823 | 8963,02128 | 65,0823154 |
| WP_002461274.1 | slg:SLGD_00877 | purine-nucleoside phosphorylase | 26243 | 6320,86045 | 24345,4757 | 3,85160785 |
| WP_002478438.1 | slg:SLGD_01896 | glucose-6-phosphate isomerase | 49762 | 59577,317 | 284525,724 | 4,77573914 |
| WP_002459765.1 | slg:SLGD_02123 | 6-carboxytetrahydropterin synthase QueD | 16300 | 4221,32729 | 12954,4042 | 3,06879882 |
| WP_002478374.1 | slg:SLGD_01794 | class I SAM-dependent rRNA methyltransferase | 44868 | 389,495216 | 8385,08809 | 21,5280901 |
| WP_002479377.1 |  | malate dehydrogenase | 33861 | 2001,87827 | 7708,94848 | 3,85085776 |
| WP_002478045.1 | slg:SLGD_01226 | bifunctional oligoribonuclease/PAP phosphatase NrnA | 35729 | 436,594784 | 5722,51947 | 13,1071641 |
| WP_012990667.1 | slg:SLGD_00154 | hypothetical protein | 50011 | 31916,8994 | 656332,236 | 20,5637844 |
| WP_002461222.1 | slg:SLGD_00924 | 3-hydroxyacyl-[acyl-carrier-protein] dehydratase FabZ | 15981 | 41956,3054 | 231844,327 | 5,52585183 |
| WP_002478042.1 | slg:SLGD_01223 | metal-dependent hydrolase | 25428 | 7436,40213 | 40638,1896 | 5,46476494 |
| WP_002477957.1 | slg:SLGD_01038 | hypothetical protein | 20391 | 5073,4446 | 142022,743 | 27,9933563 |
| WP_002479316.1 | slg:SLGD_02037 | hypothetical protein | 67634 | 1206,42502 | 20071,8135 | 16,6374314 |
| WP_002478245.1 | slg:SLGD_01564 | catalase | 57828 | 2661,7637 | 24507,5208 | 9,20724885 |
| WP_002458806.1 | slg:SLGD_01165 | 6,7-dimethyl-8-ribityllumazine synthase | 16426 | 164,67235 | 6544,80125 | 39,7443848 |
| WP_002479270.1 | slg:SLGD_02450 | LysM peptidoglycan-binding domain-containing protein | 35385 | 358060,329 | 2822055,91 | 7,88150957 |

**Supplementary Table 3.** The 20 most abundant proteins among the proteins significantly more abundant in the biofilm matrix of CC3 strain (*n* = 148) and CC6 strain (*n* = 54) strains in TSBG, listed in order of abundance for each strain. NA: not applicable

| **Uniprot accession number (WP)** | **KEGG accession number (slg)** | **Description** | **CC3 strain mean normalized abundance** |  | **Uniprot accession number (WP)** | **KEGG accession number (slg)** | **Description** | **CC6 strain mean normalized abundance** |
| --- | --- | --- | --- | --- | --- | --- | --- | --- |
| WP_002460777.1 | slg:SLGD_00206 | hypothetical protein | 3764841 |  | WP_002479270.1 | slg:SLGD_02450 | LysM peptidoglycan-binding domain-containing protein | 2822055 |
| WP_002479363.1 | slg:SLGD_02116 | LTA synthase family protein | 15037905 |  | WP_012990650.1 | NA | hypothetical protein, partial | 2452215 |
| WP_002479219.1 | slg:SLGD_02520 | hypothetical protein | 876603 |  | WP_012990667.1 | slg:SLGD_00154 | hypothetical protein | 656332 |
| WP_002460510.1 | slg:SLGD_00424 | transglycosylase | 692462 |  | WP_002479452.1 | slg:SLGD_02329 | alanine dehydrogenase | 463262 |
| WP_002460046.1 | slg:SLGD_00799 | 50S ribosomal protein L17 | 453072 |  | WP_002479246.1 | slg:SLGD_02481 | peroxiredoxin | 342630 |
| WP_002459425.1 | slg:SLGD_01782 | pyruvate dehydrogenase (acetyl-transferring) E1 component subunit alpha | 441999 |  | WP_002478438.1 | slg:SLGD_01896 | glucose-6-phosphate isomerase | 284525 |
| WP_002459695.1 | slg:SLGD_02054 | type I glyceraldehyde-3-phosphate dehydrogenase | 433876 |  | WP_002460471.1 | slg:SLGD_00449 | CHAP domain-containing protein | 257182 |
| WP_002459424.1 | slg:SLGD_01781 | alpha-ketoacid dehydrogenase subunit beta | 361847 |  | WP_012990978.1 | slg:SLGD_02252 | hypothetical protein | 242308 |
| WP_002478556.1 | slg:SLGD_00388 | fructose bisphosphate aldolase | 219062 |  | WP_002461222.1 | slg:SLGD_00924 | 3-hydroxyacyl-[acyl-carrier-protein] dehydratase FabZ | 231844 |
| WP_002478262.1 | slg:SLGD_01592 | glycerol kinase | 133189 |  | WP_002460942.1 | slg:SLGD_00048 | L-lactate dehydrogenase | 201321 |
| WP_002479252.1 | slg:SLGD_02472 | IMP dehydrogenase | 126077 |  | WP_002478255.1 | slg:SLGD_01582 | type I glutamate--ammonia ligase | 183597 |
| WP_002478388.1 | slg:SLGD_01820 | LytR family transcriptional regulator | 117916 |  | WP_002477957.1 | slg:SLGD_01038 | hypothetical protein | 142022 |
| WP_002477986.1 | slg:SLGD_01122 | peptidyl-prolyl cis-trans isomerase | 112601 |  | WP_012990863.1 | slg:SLGD_01633 | methicillin resistance protein | 96385 |
| WP_002459422.1 | slg:SLGD_01779 | dihydrolipoyl dehydrogenase | 95329 |  | WP_002478419.1 | slg:SLGD_01868 | oligoendopeptidase F | 57280 |
| WP_002459921.1 | slg:SLGD_02330 | bifunctional threonine ammonia-lyase/L-serine ammonia-lyase TdcB | 87147 |  | WP_002459884.1 | slg:SLGD_02298 | heme-dependent peroxidase | 49666 |
| WP_002459694.1 | slg:SLGD_02053 | phosphoglycerate kinase | 84093 |  | WP_002478368.1 | slg:SLGD_01786 | RNase J family beta-CASP ribonuclease | 48409 |
| WP_002478832.1 | slg:SLGD_00787 | 50S ribosomal protein L6 | 74030 |  | WP_002478042.1 | slg:SLGD_01223 | metal-dependent hydrolase | 40638 |
| WP_002479198.1 | slg:SLGD_02554 | serine--tRNA ligase | 71490 |  | WP_002477992.1 | slg:SLGD_01128 | hypothetical protein | 37139 |
| WP_002478985.1 | slg:SLGD_00302 | CHAP domain-containing protein | 66476 |  | WP_002459545.1 | slg:SLGD_01902 | Glu/Leu/Phe/Val dehydrogenase | 30638 |
| WP_002459693.1 | slg:SLGD_02052 | triose-phosphate isomerase | 61208 |  | WP_002478245.1 | slg:SLGD_01564 | catalase | 24507 |

## Supplementary Figure


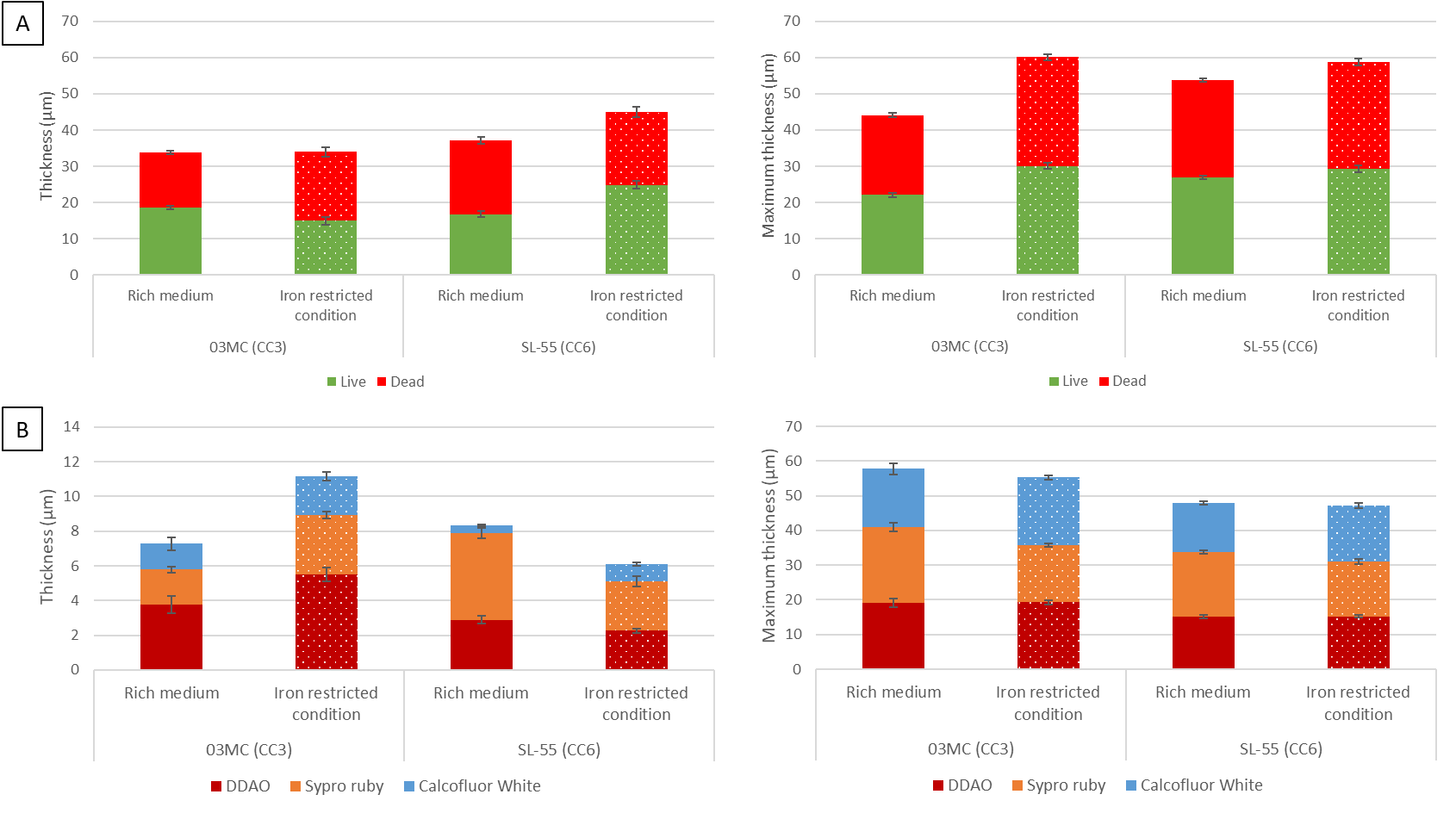


**Supplementary Figure 1.** COMSTAT image analysis of biofilm composition of living (green) and dead (red) cells (A) and biofilm matrix composition in eDNA, proteins, and polysaccharides labelling by DDAO, Sypro ruby and Calcofluor White, respectively (B) in rich medium and iron restricted medium. Data are the results of the analysis of 10 views from three independent experiments.
